# A prospective observational study with dose volume parameters predicting rectosigmoidoscopic findings and late rectosigmoid bleeding in patients with uterine cervical cancer treated by definitive radiotherapy

**DOI:** 10.1186/1748-717X-8-28

**Published:** 2013-01-31

**Authors:** Tae Hyun Kim, Joo-Young Kim, Dae Kyung Sohn, Yeon-Joo Kim, Yoon-Seok Lee, Sung Ho Moon, Sang Soo Kim, Dae Yong Kim

**Affiliations:** 1 Research Institute and Hospital, National Cancer Center, Goyang, Gyeonggi, South Korea

**Keywords:** Dose-volumetric parameters, Rectosigmoid mucosal change, Late rectosigmoid complication, Uterine cervical cancer

## Abstract

**Purpose:**

We assessed the value of dose-volumetric parameters predicting rectosigmoid mucosal changes (RMC) and late rectosigmoid complications (LRC).

**Methods:**

Between January 2004 and February 2006, 77 patients with stage IB-IIIB cervical cancer underwent external beam radiotherapy and computed tomography (CT)-based intracavitary irradiation. Total dose to the rectal point and several dose-volumetric parameters for rectosigmoid colon (D_20cc_, D_15cc_, D_10cc_, D_5cc_, D_2cc_, D_1cc_, and D_0.1cc ,_ defined as the minimal doses received by the highest irradiated volumes of 20, 15, 10, 5, 2, 1, and 0.1 cc, respectively), were calculated using the equivalent dose in 2 Gy fractions (α/β = 3, Gy_3_). The RMC and LRC were graded by rectosigmoidoscopy and Radiation Therapy Oncology Group criteria every 6 months, respectively.

**Results:**

Of 77 patients, 27 (35.1%) patients developed RMC ≥ score 3 and 22 (28.6 %) patients developed LRC ≥ grade 2. There was a positive correlation between RMC score and LRC grade (*r* = 0.728, *p* < 0.001). In multivariate analyses, D_5cc_, among the dose-volumetric parameters, was significant parameter for the risks of RMC ≥ score 3 and LRC ≥ grade 2 (*p <* 0.05).

**Conclusions:**

D_5cc_ may be a more reliable estimate than other dose-volumetric parameters for predicting the risk of RMC ≥ score 3 and LRC ≥ grade 2 in CT-based brachytherapy.

## Introduction

Traditionally, the rectal point, defined by the International Commission on Radiation Units and Measurements Report 38 (ICRU 38), has been used as a reference point to represent the rectal dose in brachytherapy of cervical cancer. However, a single point dose using two-dimensional orthogonal radiographs does not account for the exact tumor and normal tissue anatomy and is not accurate enough in estimating the risk of late rectal complication (LRC). Recently, with the introduction of three-dimensional (3D) treatment planning using computed tomography (CT) and magnetic resonance imaging (MRI), several dose-volumetric parameters, including D_2cc_, D_1cc_, and D_0.1cc_ (the minimal doses received by the highest irradiated 2 cc, 1 cc and 0.1 cc volumes of the rectum and sigmoid colon, respectively) are more frequently used in optimizing the treatment [1-6].

We have been using CT-based brachytherapy since 2004 before we moved into MRI-based brachytherapy in 2008 [7]. A prospective observational study to assess the value of dose-volumetric parameters predicting rectosigmoid mucosal changes (RMCs) using serial rectosigmoidoscopy was started for the patients treated with 3D CT-based brachytherapy. A preliminary result showed that the aforementioned dose-volumetric parameters were significantly associated with RMC on rectosigmoidoscopy at 12 months [8]. The current report is our final result of the study showing the correlations among RMC, LRC, and dose-volumetric parameters.

## Methods

### Patients

Between January 2004 and February 2006, a total of 80 patients who were treated with definitive radiotherapy (RT) were enrolled in this study. The eligibility criteria were described previously [8]. The study consisted of a specific interview about the rectal symptom and one rectosigmoidoscopy every 6 months for 2 years. The study was approved by our institutional review board and all patients provided written informed consent. Of 80 patients, three patients who died within 24 months due to local and/or distant disease progression after RT were excluded from analysis; the remaining 77 patients were analyzed. Prior to RT, all patients underwent a pelvic examination, chest radiography, cystoscopy, rectosigmoidoscopy, and pelvic MRI with/without CT. Patient characteristics are summarized in Table 1.

**Table 1:** Associations of clinical and dose-volumetric parameters parameters with the rectosigmoid mucosal change score (RMC) ≥ 3 and late rectal complication (LRC) grade ≥ 2

		**Total**	**RMC**			**LRC**		
		**Patients, n (%)**	**Score < 3, n (%)**	**≥ Score 3, n (%)**	***p*-value**	**Grade < 2, n (%)**	**Grade ≥ 3, n (%)**	***p*-value**
Age (years)	≤ 60	50 (64.9)	35 (70)	15 (30)	0.222^*^	38 (76)	12 (24)	0.292^*^
	> 60	27 (35.1)	15 (55.6)	12 (44.4)		17 (63)	10 (37)	
Histology	Squamous cell carcinoma	70 (90.9)	44 (62.9)	26 (37.1)	0.411^*^	49 (70)	21 (30)	0.666^*^
	Adenocarcinoma	7 (9.1)	6 (85.7)	1 (14.3)		6 (85.7)	1 (14.3)	
Tumor size (cm)	≤ 4	41 (53.2)	29 (70.7)	12 (29.3)	0.339^*^	32 (78)	9 (22)	0.210^*^
	> 4	36 (46.8)	21 (58.3)	15 (41.7)		23 (63.9)	13 (36.1)	
Stage	IB-IIA	32 (41.6)	27 (84.4)	5 (15.6)	0.003^*^	29 (90.6)	3 (9.4)	0.002^*^
	IIB-IIIB	45 (58.4)	23 (51.1)	22 (48.9)		26 (57.8)	19 (42.2)	
Concurrent chemotherapy	Yes	66 (85.7)	43 (65.2)	23 (34.8)	1.000^*^	48 (72.7)	18 (27.3)	0.719^*^
	No	11 (14.3)	7 (63.6)	4 (36.4)		7 (63.6)	4 (36.4)	
Diabetes Mellitus	Yes	6 (7.8)	5 (83.3)	1 (16.7)	0.417^*^	5 (83.3)	1 (16.7)	0.668^*^
	No	71 (92.2)	45 (16.7)	26 (44.4)		50 (70.4)	21 (29.6)	
Hypertension	Yes	20 (26)	10 (50)	10 (50)	0.172^*^	12 (60)	8 (40)	0.251^*^
	No	57 (74)	40 (70.2)	17 (29.8)		43 (75.4)	14 (24.5)	
Smoking	Yes	12 (15.6)	10 (83.3)	2 (16.7)	0.491^*^	9 (75)	3 (25)	0.524^*^
	No	65 (84.4)	45 (69.2)	20 (30.8)		41 (63.1)	24 (36.9)	
D_RP_ (Gy_3_)	(μ ± σ)		69.1 ± 12.3	75.4 ± 18.3	0.124^†^	69.2 ± 11.9	76.7 ± 20.0	0.120^†^
D_0.1cc_ (Gy_3_)	(μ ± σ)		87.2 ± 15.6	101.7 ± 26.7	0.014^†^	87.9 ± 15.9	103.1 ± 28.4	0.026^†^
D_1cc_ (Gy_3_)	(μ ± σ)		75.1 ± 10.9	85.2 ± 14.4	0.001^†^	75.8 ± 11.1	85.6 ± 15.2	0.002^†^
D_2cc_ (Gy_3_)	(μ ± σ)		70.8 ± 9.8	79.6 ± 11.5	0.001^†^	71.5 ± 9.9	79.9 ± 12.0	0.002^†^
D_5cc_ (Gy_3_)	(μ ± σ)		64.6 ± 8.2	72.0 ± 8.4	< 0.001^†^	65.2 ± 8.3	72.2 ± 8.8	0.002^†^
D_10cc_ (Gy_3_)	(μ ± σ)		59.5 ± 6.4	63.4 ± 6.0	0.011^†^	60.1 ± 6.7	62.7 ± 5.8	0.107^†^
D_15cc_ (Gy_3_)	(μ ± σ)		56.3 ± 5.8	60.2 ± 5.0	0.004^†^	56.8 ± 5.8	59.9 ± 5.3	0.034^†^
D_20cc_ (Gy_3_)	(μ ± σ)		55.4 ± 5.8	59.1 ± 4.8	0.006^†^	55.9 ± 5.9	58.8 ± 4.9	0.040^†^

Abbreviations: D_RP_ = doses to the reference points for the rectum; D_0.1cc_, D_1cc_, D_2cc_, D_5cc_, D_10cc_, D_15cc_, and D_20cc_ = minimal doses received by the highest irradiated 0.1, 1, 2, 5, 10, 15, and 20 cc volumes of the rectosigmoid colon, respectively.; μ = mean; σ = standard deviation.

^*^Fisher’s exact test.

^†^ Student’s *t*-test.

### Treatment

RT consisted of a combination of external beam radiation therapy (EBRT) and high dose rate ICR (HDR-ICR). The details of the RT techniques have been described [7,8]. In brief, EBRT was delivered by a linear accelerator with a 15-MV X-ray using the 4-field box technique in daily fractions of 1.8–2 Gy, 5 days/week, with a total parametrial dose of 45–66 Gy (median, 54 Gy). A 4 cm width midline shielding was placed at 36 Gy in 4 elderly patients with stage Ib1 diseases, at 39.6 Gy in one patient with Stage IIA, and at 45 Gy in 46 patients with stage Ib1 to IIB. Whole pelvis was treated up to 50.4 Gy for the rest of the patients without midline block. HDR-ICR was delivered in 3.3–5 Gy/fraction twice a week up to a median dose of 29 Gy (range, 20–35 Gy) prescribed to the point A using a ^192^Ir remotely controlled afterloading system. Sixty-six patients received concurrent chemotherapy as follows: cisplatin (5 cycles of weekly intravenous injection at 40 mg/m^2^/day) in 65 patients and cisplatin plus 5–fluorouracil (3 cycles of intravenous injection of cisplatin at 50 mg/m^2^/day, day 1, and 1000 mg/m^2^/day of 5-fluorouracil, days 1–5, followed by 14 days rest at each cycle) in one patient.

The imaging, contouring, and planning details were described previously [8]. In brief, a series of transverse images of the pelvic region were acquired using a CT simulator with the applicators in place. The target volume and organs at risk (OARs) were delineated by radiation oncologists. All patients were examined by a baseline MRI of the pelvis and an additional MRI prior to ICR simulation to aid contouring of clinical target volume (CTV). CTV included the whole cervix plus any residual parametrial disease and was in accordance with high-risk CTV defined by the Gynecologic Groupe Européen de Curiethérapie and the European Society for Therapeutic Radiology and Oncology (GEC-ESTRO) recommendations [6]. Outer organ contours were delineated for the bladder and rectosigmoid colon; the rectum was defined from the anorectal junction to the rectosigmoid flexure. Treatment planning was optimized from January 2005 with the goal that the dose receiving ≥90% of CTV must be greater than the prescribed dose and that the volume treated with at least the prescribed dose must be ≥90%. The rectum and sigmoid colon should each receive <90% of the prescribed dose at any point.

### Determination of dose-volume parameters

Cumulative DVHs for each OAR were computed and the D_20cc_, D_15cc_, D_10cc_, D_5cc_, D_2cc_, D_1cc_, and D_0.1cc_ of each OAR (the minimal doses received by the highest irradiated 20 cc, 15 cc, 10 cc, 5 cc, 2 cc, 1 cc, and 0.1 cc volumes of OAR, respectively) were determined. The doses to point A (D_point A_) and ICRU reference points for the bladder and rectum (D_BP_ and D_RP_, respectively) were also calculated. The total dose (EBRT plus ICR) was calculated as the biologically equivalent dose in 2-Gy fractions (EQD2) using the linear quadratic model [9]. The equation used to calculate the total EQD2 was:

(1) $$\begin{array}{c} {\text{EQD}2}_{\mathrm{total}}={\mathrm{EQD}2}_{\mathrm{EBRT}}+{\mathrm{EQD}2}_{\mathrm{ICR}}\\ \;=\mathrm{Nd}\left[\left(d+\alpha /\beta \right)/\left(2+\alpha /\beta \right)\right]\\ \;+N_{B}d_{B}\left[\left(d_{B}+\alpha /\beta \right)/\left(2+\alpha /\beta \right)\right] \end{array}$$

where Nd is the total dose of EBRT (before central shielding), d is the fractional dose of EBRT, N_B_d_B_ is the total dose of HDR-ICR, and d_B_ is the fractional dose of HDR-ICR to the CTV and OARs. The α/β values of 10 and 3 were applied for the CTV and OARs, respectively.

### Evaluation of LRCs and follow-up

Patient follow-up was performed every 3 months in the first 2 years following RT, every 4 months in the third year, then every 6 months for up to 5 years, and yearly thereafter. Apart from the routine evaluation of the status of cervical cancer [8], the follow-up included a specific interview evaluating the symptom complex related to the rectal morbidity (i.e., bleeding) and a flexible rectosigmoidoscopy (CF-Q240 or CF-H260; Olympus Optical Co., Tokyo, Japan) examination at every 6 months for the first 2 years. In total, rectosigmoidoscopy was performed in 49 patients at 6, 12, 18, and 24 months; 12 patients at 6, 12, and 18 months; 9 patients at 6, 12, and 24 months; and 7 patients at 6 and 12 months. For evaluating RMC, we adopted an endoscopic scoring system [8,10] and determined the worst score through the whole wall of the rectosigmoid colon among all serial examinations. LRC was classified according to the Radiation Therapy Oncology Group (RTOG) late radiation morbidity scoring criteria [11].

### Statistical analysis

To analyze dose-volumetric effects, an RMC ≥ score 3 and LRC ≥ grade 2 were used as quantal endpoints. Correlations among RMC, LRC, clinical, and dose-volumetric parameters were assessed using Fisher’s exact test, Student’s *t*-test, and Spearman’s and Pearson’s correlation coefficient tests, respectively. The overall survival rate was calculated using the Kaplan-Meier method; all time intervals were measured from the first day of RT. Dose–response relationships influencing the probabilities of RMC ≥ scores 3, LRC ≥ grades 2 were analyzed using a logistic regression model. In multivariate analysis, a stepwise logistic regression model was used with all clinical and dose-volumetric parameters to predicting the risks of RMC and LRC. In this procedure, the forward selection of the parameter was processed by the score chi-square test and the backward elimination by the Wald test. All statistical tests were 2-sided and were performed using STATA software (version 9.0, Stata Corp.; College Station, TX). Values of *p* <0.05 indicated statistical significance.

## Results

The median follow-up and 5-year actuarial overall survival rate for all patients were 70.8 months (range, 24.4–83.9 months) and 90.9%, respectively. The mean CTV for ICR was 48.2 ± 20.3 cm^3^ and the volume treated with at least the prescribed dose was 94 ± 7%. The mean volume of bladder, rectum, and sigmoid colon was 197.9 ± 104.6, 79.5 ± 31.5, and 30.8 ± 24.9 cm^3^, respectively. The mean EQD2 data were comparable with data from other centers (Additional file 1: Table S1), [1,12].

**Figure 1:**
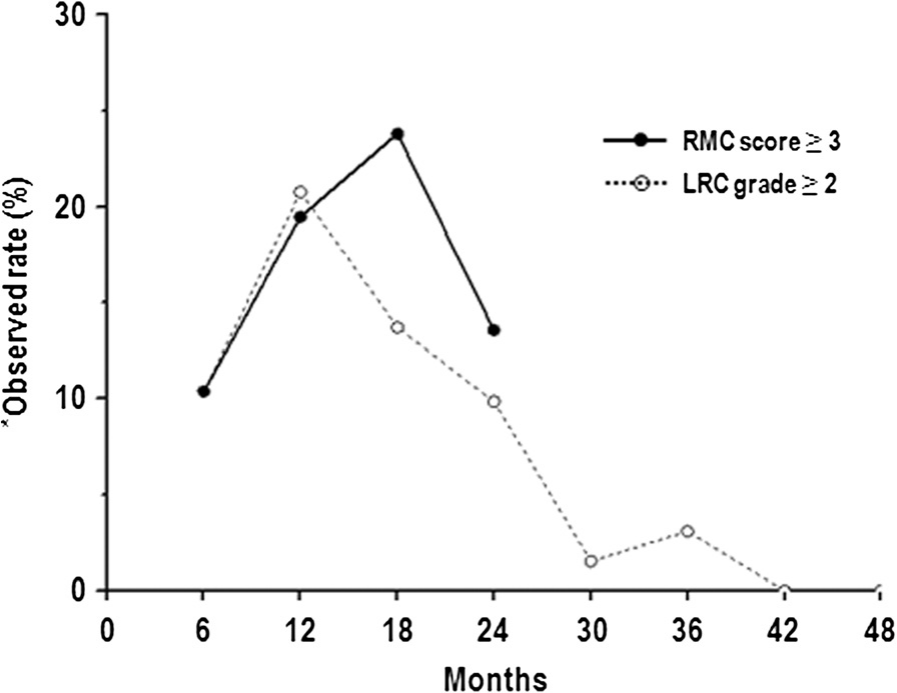
Observed rate of a rectosigmoid mucosal change (RMC) score ≥3 and late rectal complication (LRC) grade ≥2.^*^Observed rate = number of patients who developed an RMC score ≥ 3 or LRC grade ≥ 2 / number of patients at risk at a specific time point (%).

According to the endoscopic scoring criteria, overall distribution of the worst RMC scores through the rectosigmoid colon was score 1 in 30 patients, score 2 in 20 patients, score 3 in 22 patients, score 4 in 1 patient, and score 5 in 4 patients. According to RTOG grade, the overall distribution of LRC grades was grade 0 in 40 patients, grade 1 in 15 patients, grade 2 in 21 patients, grade 3 in 0 patients, grade 4 in 1 patient, and grade 5 in 0 patients. The median interval from the start date of RT to the onset of LRC was 14.6 months (range, 2.6–49.9 months). LRC developed within 24 months in 30 (81.1%) patients. For the rest of the patients (n = 7), LRC of RTOG grade 1–2 developed between the period of 25 ~ 50Mo). Figure 1 shows a concordant pattern in observed rates of RMC ≥ score 3 and LRC ≥ grade 2 at serial time points and there was a positive correlation between RMC score and LRC grade (*r* =0.728, *p* < 0.001), (Additional file 1: Table S2).

The results of an univariate analysis evaluating the associations of clinical and dose-volumetric parameters with RMC ≥ score 3 and LRC ≥ grade 2 are summarized in Table 1. Among the clinical parameters, only stage was found to be significantly associated with the risk of RMC ≥ score 3 and LRC ≥ grade 2 (*p* < 0.05). The mean values of all dose-volumetric parameters, except in D_0.1cc_, in advanced stage (IIB-IIIB) were significantly higher than those in earlier stage (IB-IIA) (D_RP_: 74.3 ± 15.6 vs. 67 ± 12.8 Gy_3_, *p =* 0.036; D_0.1cc_: 95.6 ± 22.7 vs. 87.6 ± 18.1 Gy_3_, *p =* 0.103; D_1cc_: 81.7 ± 13.1 vs. 74.4 ± 12.1 Gy_3_, *p =* 0.015; D_2cc_: 76.7 ± 10.7 vs. 69.9 ± 10.6 Gy_3_, *p =* 0.007; D_5cc_: 69.8 ± 8.4 vs. 63.5 ± 8.5 Gy_3_, *p =* 0.002; D_10cc_: 63.2 ± 5.7 vs. 57.5 ± 6.2 Gy_3_, *p <* 0.001; D_15cc_: 59.8 ± 5.1 vs. 54.8 ± 5.5 Gy_3_, *p <* 0.001; and D_20cc_: 58.8 ± 5.1 vs. 53.8 ± 5.4 Gy_3_, *p <* 0.001). Of the dose-volumetric parameters, the mean values of D_0.1cc_, D_1cc_, D_2cc_, D_5cc_, D_15cc_, and D_20cc_, but not of D_RP_ for both RMC and LRC and D_10cc_ for LRC, in patients who developed an RMC ≥ score 3 or LRC ≥ grade 2 were found to be significantly higher than those in patients who did not (*p* < 0.05) (Table 1). The dose-volumetric parameters were all closely interrelated (D_5cc_ vs. D_20cc_: *r* = 0.835, *p <* 0.001; D_5cc_ vs. D_15cc_: *r* = 0.837, *p <* 0.001; D_5cc_ vs. D_10cc_: *r* = 0.782, *p <* 0.001; D_5cc_ vs. D_2cc_: *r* = 0.974, *p <* 0.001; D_5cc_ vs. D_1cc_: *r* = 0.934, *p <* 0.001; D_5cc_ vs. D_0.1cc_: *r* = 0.795, *p <* 0.001; and D_5cc_ vs. D_RP_: *r* = 0.555, *p <* 0.001, values only for D_5cc_ were described).

**Figure 2:**
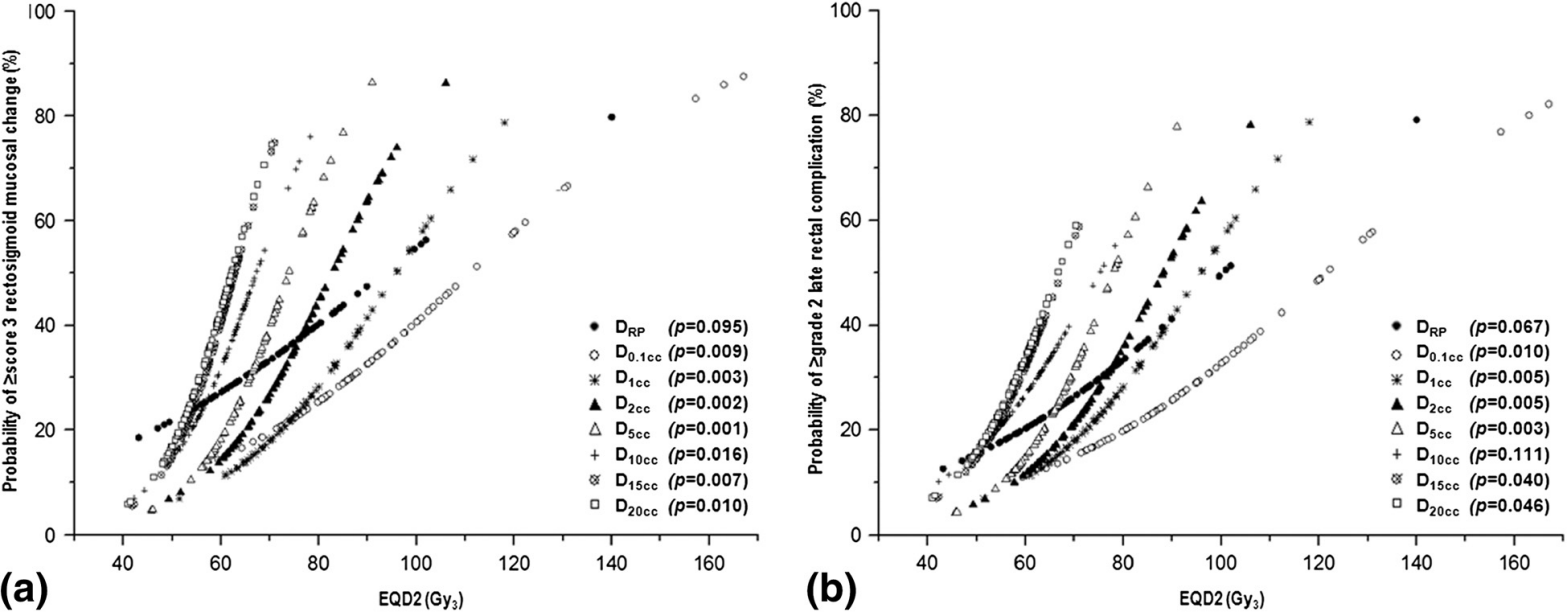
**Estimated probability of a rectosigmoid mucosal change (RMC) score ≥3 (a) and late rectal complication (LRC) grade ≥2 (b) according to the dose-volumetric parameters.** The probability values are based on a logistic regression analysis. Abbreviations: same as in Table 2.

**Figure 3:**
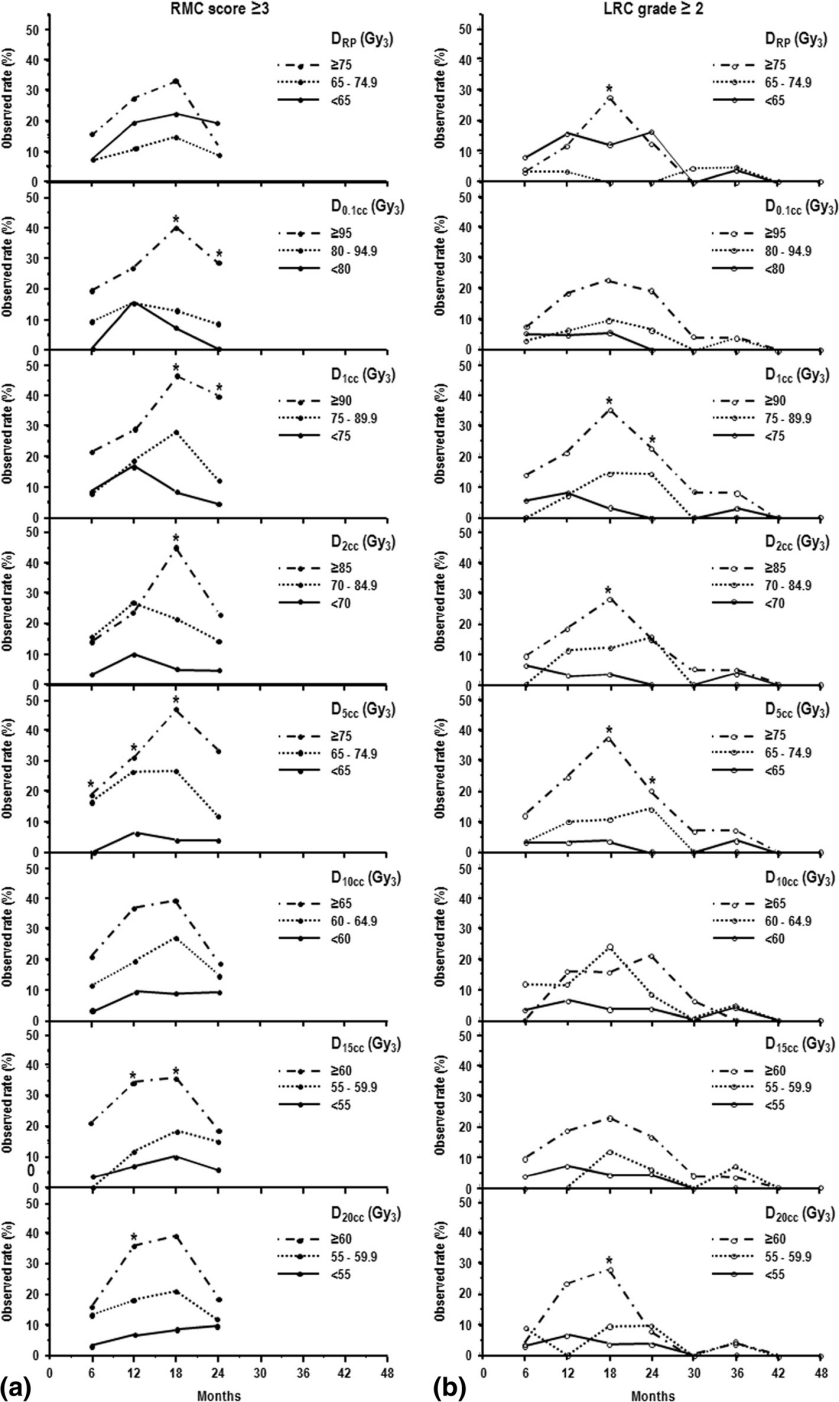
**Observed rate of a rectosigmoid mucosal change (RMC) score ≥3 (a) and late rectal complication (LRC) grade ≥2 (b) according to the dose-volumetric parameters.** Abbreviations: same as in Table 2 and Figure 1. ^*^p < 0.05, Fisher’s exact test.

The estimated probability values for RMC ≥ score 3 and LRC ≥ grade 2 according to the dose-volumetric parameters are depicted in Figure 2a and b, respectively. The probability curves of all dose-volumetric parameters, except in D_RP_, had a statistical significance and the range of radiation dose in the probability curve was smallest for D_20cc_ and greatest for D_0.1cc_. We chose two individual cutoff points for each dose-volumetric parameter considering similar size among the subgroups and changes in the curve gradients and then evaluated the effects of the dose-volumetric parameters on the risk of RMC ≥ score 3 and LRC ≥ grade 2 (Table 2). The cumulative incidence of RMC ≥ score 3 was significantly different among the subgroups according to D_0.1cc_, D_1cc_, D_2cc_, D_5cc_, D_10cc_, D_15cc_, and D_20cc_ (*p* < 0.05), in contrast to those according to D_RP_ (*p* > 0.05). The cumulative incidence of LRC ≥ grade 2 was significantly different among the subgroups according to D_1cc_, D_2cc_, D_5cc_, and D_15cc_, in contrast to those in subgroups according to the D_RP_, D_0.1cc_, D_10cc_, and D_20cc_ (*p >* 0.05). The observed rate of RMC ≥ score 3 and LRC ≥ grade 2, at every 6 month intervals, according to dose-volumetric parameters are depicted in Figure 3a and b, respectively. With increasing the dose-volumetric parameters, both RMC ≥ score 3 and LRC ≥ grade 2 rates showed an increasing trend, with those differences being the most prominent at 18 months (Figure 3). In multivariate analysis with all clinical and dose-volumetric parameters, D_5cc_ remained as a significant factor than other dose-volumetric parameters for the risk of RMC ≥ score 3 (*p* < 0.05) and the risk of LRC ≥ grade 2 along with age and stage, and age, respectively (*p <* 0.05) (Table 3).

**Table 2:** Cumulative incidence of a rectosigmoid mucosal change (RMC) score ≥ 3 and late rectal complication (LRC) grade ≥ 2 in relation to various dose-volumetric parameters receiving three different dose subgroups

**Parameters**		Incidence^*^of ≥ score 3 RMC	^**†**^***p*-value**	Incidence^*^of ≥ grade 2 LRC	^**†**^***p*-value**
D_RP_ (Gy_3_)	< 65	8/25 (32.0)	0.260	7/25 (28.0)	0.070
	65–74.9	7/27 (25.9)		4/27 (14.8)	
	≥ 75	12/25 (48.0)		11/25(44.0)	
D_0.1cc_ (Gy_3_)	< 80	4/19 (21.1)	0.046	2/19 (10.5)	0.066
	80–94.9	9/32 (28.1)		9/32 (28.1)	
	≥ 95	14/26 (53.9)		11/26 (42.3)	
D_1cc_ (Gy_3_)	< 75	8/36 (22.2)	0.021	6/36 (16.7)	0.021
	75–89.9	10/27 (37.0)		8/27 (29.6)	
	≥ 90	9/14 (64.3)		8/14 (57.1)	
D_2cc_ (Gy_3_)	< 70	5/30 (16.7)	0.010	4/30 (13.3)	0.041
	70–84.9	10/26 (38.5)		9/26 (34.6)	
	≥ 85	12/21 (57.1)		9/21 (42.9)	
D_5cc_ (Gy_3_)	< 65	4/31(12.9)	0.002	3/31 (9.7)	0.002
	65–74.9	13/30 (43.3)		10/30 (33.3)	
	≥ 75	10/16 (62.5)		9/16 (56.3)	
D_10cc_ (Gy_3_)	< 60	6/32 (18.8)	0.017	5/32 (15.6)	0.082
	60–64.9	10/26 (38.5)		9/26 (34.6)	
	≥ 65	11/19 (57.9)		8/19 (42.1)	
D_15cc_ (Gy_3_)	< 55	4/28 (14.3)	0.006	4/28 (14.3)	0.036
	55–59.9	6/17 (35.3)		4/17 (23.5)	
	≥ 60	17/32 (53.1)		14/32 (43.8)	
D_20cc_ (Gy_3_)	< 55	5/30 (16.7)	0.011	5/30 (16.7)	0.092
	55–59.9	8/22 (36.4)		6/22 (27.3)	
	≥ 60	14/25 (56.0)		11/25 (44.0)	

Abbreviations: same as in Table 2.

^*^Cumulative incidence = number of patients who developed an RMC score ≥ 3 or LRC grade ≥ 2 / number of patients at risk (%) during follow-up.

^†^Fisher’s exact test.

**Table 3:** Multivariate analysis of factors influencing a rectosigmoid mucosal change (RMC) score ≥ 3 and late rectal complication (LRC) grade ≥ 2

			**RMC**			**LRC**	
**Factor**		**Odd Ratio**	**95% CI**	*p*-value^*^	**Odd Ratio**	**95% CI**	*p*-value^*^
Age (years)	≤ 60	1.000	–	0.008	–	–	NS
	> 60	21.200	2.233–201.247		–	–	
Stage	IB-IIA	1.000	–	0.006	1.000	–	0.017
	IIB-IIIB	24.084	2.543–228.124		5.426	1.359–21.657	
D_5cc_ (Gy_3_)	< 65	1.000	–	0.013	1.000	–	0.031
	65–74.9	22.426	2.329–215.962		4.222	0.976–18.259	
	≥ 75	31.103	3.084–313.657		8.528	1.710–42.544	

Abbreviations: CI = confidence interval; NS = not significant; the others abbreviations are as in Table 2.

^*^Multivariate logistic regression analysis.

## Discussion

The D_RP_, RP ratio (D_RP_/D_Point A_), and maximal rectal point dose were used to evaluate the risk of LRC traditionally [11,13-17]. We previously showed that the biologically effective dose at the ICRU rectal point (≤125 vs. >125 Gy_3_) calculated at the orthogonal film-based brachytherapy was equivalent to an EQD2 of 75 Gy_3_, and was significantly associated with a 5-year actual risk of an LRC grade ≥2 (5.4 vs. 36.1%; *p* < 0.001) [11]. Starting with the 3D CT-based brachytherapy in 2004, a prospective observational study was designed to estimate the value of dose-volume parameters in predicting the risk of RMC and LRC. Our preliminary analysis showed that several dose-volumetric parameters (D_0.1cc_, D_1cc_, D_2cc_, and D_RP_) were significantly associated with the risk of an RMC ≥ score 2 at 12 months [8]. As a continuum study with longer follow-up time, the present data demonstrated close correlations among RMC, LRC, and dose-volumetric parameters. Our data also suggests that D_5cc_ may be a more reliable estimate than other dose-volumetric parameters for predicting the risk of an RMC ≥ score 3 and LRC ≥ grade 2.

Conceptually, dose wall histograms (DWHs) are potentially more valuable than DVHs in determining radio-biological effects on tubular structures. However, it is difficult to obtain DWHs in clinical practice because of the major uncertainties which may result from the very small dimensions of the organs and the inability to have automatically-generated second contours at selected distances by the treatment planning system. Thus, it was allowed to obtain DVHs for the rectosigmoid colon using an external organ contouring method in the GEC-ESTRO recommendations [2]. Apart from the contouring method of OAR, several previous data have demonstrated that doses for ≤2 cc volume of rectum (i.e., D_2cc_ ,D_1cc_ , and D_0.1cc_) is a reliable and consistent factors for predicting the risk of LRC rather than those for ≥5 cc volume of rectum (i.e., D_5cc_ , and D_10cc_ ) [4,18]. On this background, GEC-ESTRO has recommended that D_2cc_, D_1cc_, D_0.1cc_, and D_RP_ are mandatory for dose report for OAR [1-5], but the report on the D_5cc_ and D_10cc_ was left optional. There is a paucity of study which examined the value of D_5cc_ for predicting the risk of LRC since the guideline was set up by GEC-ESTRO.

There are several suggestions which may aid in support our result that D_5cc_ was shown to be the most powerful factor predicting both RMC and LRC. First, the rectum is quite often located asymmetrically or deviated to perpendicular line from the cervical os and ICR applicator, and the dose to the smaller volume can over- or underestimate the maximal dose of the rectum as the point doses do in this situation [4]. Second, dose-volumetric parameters have inherent uncertainties from interfractional setup variability of the ICR applicator and the position of the hollow organs in relation to the changes of tumor volumes over time. In Vienna University, the patients were asked to empty rectosigmoid colon before ICR and 3D image-based treatment planning was performed at every ICR fraction for four fractions [4,5,19], whereas in our institute, bowel emptying was not a requirement before ICR and 3D image-based treatment planning was performed only once under the assumption that the dose-volume parameters calculated from an initial ICR plan was the same throughout the rest of procedures. Our practice pattern could have provided higher chance of interfractional variability than the Vienna’s one. This is a limitation of our study and thus the interpretation and comparison of our data with other studies in the literatures should be careful. However, our data could be valuable reference for the institutes where fractionated regimen is used on outpatient basis and 3D brachytherapy plan cannot be performed at every treatment session.

One of the caveats of our study is that only 63.6% of the patients completed all 4 examinations although our study intended 4 rectosigmoidoscopic examinations every 6 months for the first 2 years; the rest (36.4%) completed only 2–3 examinations. Although 80% of the LRC occurred within 24 months, there is about 20% (n = 7) of the LRC events still developed after 24 months (range, 25 – 50 months). This may raise questions that our 2-year RMC data may not enough to predict the risk for LRC. However, this does not seem to be the case because both RMC and LRC scores showed a consistent time pattern with the peak of the worst score develops at 18 months post-RT and then declines (Figure 3). Ippolito et al. [20] showed that RMC using rectosigmoidoscopy at 1 year after completion of RT in 101 prostate cancer patients had a good positive correlation with clinical LRC, suggesting that RMC of high grade precedes clinical rectal bleeding, which most commonly occurs at 1 ~ 2 years post-radiotherapy. Georg et al. [5] also showed that most significant RMC matches to the area of the highest radiation dose in 35 cervical cancer patients, however, it could not be elucidated in that study at what time point is the rectosigmoidoscopy most effective in predicting LRC because of the variable time of the endoscopic examination. Our present study shows that the most severe degree of RMC occurs at 18 months post-RT, which is probably the same time point of the median time of development of LRC (15 month in our series). From our and others’ results [8,20], post-RT 24 month may be an enough time to predict most of the events of LRC.

There are controversial results in which the risk of LRC could be influenced by various clinical parameters, such as age, diabetes, hypertension, smoking, and stage [14,21-24]. Multivariate analysis of the present study showed that both age and stage was significantly associated with the risk of RMC and LRC, respectively (Table 3), but no association was detected between the RMC/LRC and other parameters. We consider that this finding may be due to the small patient number of our study, and these parameters still need to be investigated further.

## Conclusion

In conclusion, we showed positive correlations among the examined dose-volumetric parameters, RMC, and LRC and suggested that that D_5cc_ might be a more reliable estimate for predicting the risk of RMC ≥ score 3 and LRC ≥ grade 2 than the other dose-volumetric parameters although D_1cc_ and D_2cc_ had also significance for with the risk of RMC and LRC in univariate analysis. Given that CT-based brachytherapy is more assessable technique [25-28] globally than the MRI-based one because of the problems of logistics and resources, our result may be meaningful in clinical practice and should be verified by further studies in broader societies using 3D image-based brachytherapy, especially in the institutes where fractionated regimen is used on outpatient basis.

## Competing interests

None of the authors have potential conflicts of interest.

## Authors’ contributions

JK and THK are responsible for the study design. JK, THK, DKS, YK, YL, SHM, SSK, and DYK collected the clinical data and drafted the manuscript. THK, YK, and YL revised the manuscript. DKS and YK collected the pathologic data and analysis. JK, THK, YK, YL, SHM, SSK, and DYK were responsible for the treatment and evaluation of the patients. JK, DYK and THK provided oversight of the analysis of data and reviewing of the manuscript. All authors read and approved the final manuscript.

## Supplementary Material

Additional file 1: Table S1 Mean values of the biological dose for point A, clinical target volume (CTV), and organs at risk. Table S2. Correlation between the rectosigmoid mucosal change (RMC) score and late rectal complication (LRC) grade. Click here for file
