# Supplementary material for: A prospective observational study with dose volume parameters predicting rectosigmoidoscopic findings and late rectosigmoid bleeding in patients with uterine cervical cancer treated by definitive radiotherapy
Source: Radiat Oncol. 2013 Jan 31;8:28. doi: 10.1186/1748-717X-8-28 (PMC3570440; doi:10.1186/1748-717X-8-28)
Supplement: Additional file 1: Table S1 — Mean values of the biological dose for point A, clinical target volume (CTV), and organs at risk. Table S2. Correlation between the rectosigmoid mucosal change (RMC) score and late rectal complication (LRC) grade. [file 1748-717X-8-28-S1.doc]

Table s1. Mean values of the biological dose for point A, clinical target volume (CTV), and organs at risk

|  | EQD2 (Gy_10_ or Gy_3_, μ ± σ) | | |
| --- | --- | --- | --- |
|  | ^*^Our study | ^*^Kirisits et al. | ^†^Lang et al. |
| D_Point A_ | 78.6 ± 7.3 | 82 ± 9 | 87.0 ± 3.5 |
| Right | 78.6 ± 7.2 | - | 89.0 ± 4.6 |
| Left | 78.6 ± 7.4 | - | 84.3 ± 2.5 |
| CTV |  |  |  |
| D_100_ | 67.5 ± 7.8 | 66 ± 7 | 67.7 ± 5.5 |
| D_90_ | 85.3 ± 10.1 | 87 ± 10 | 86.0 ± 1.0 |
| Bladder |  |  |  |
| D_BP_ | 78.2 ± 20.3 | 75 ± 16 | 76.3 ± 20 |
| Rectum |  |  |  |
| D_RP_ | 71.3 ± 14.8 | 69 ± 13 | 61.7 ± 3.5 |
| D_0.1cc_ | 81.8 ± 16.7 | 77 ± 10 | 66.7 ± 9.0 |
| D_1cc_ | 72.3 ± 11.6 | 66 ± 7 | - |
| D_2cc_ | 68.7 ± 10.2 | 64 ± 6 | 59.7 ± 5.9 |
| D_5cc_ | 63.7 ± 8.4 | - | 55.7 ± 4.2 |
| Sigmoid |  |  |  |
| D_0.1cc_ | 85.0 ± 22.5 | 79 ± 12 | 85.7 ± 6.4 |
| D_1cc_ | 73.4 ± 14.5 | 67 ± 8 | - |
| D_2cc_ | 69.2 ± 12.5 | 63 ± 7 | 63.3 ± 3.5 |
| D_5cc_ | 63.1 ± 10.5 | - | 54.7 ± 2.1 |
| Rectosigmoid |  |  |  |
| D_0.1cc_ | 92.3 ± 21.2 | - | - |
| D_1cc_ | 78.6 ± 13.1 | - | - |
| D_2cc_ | 73.9 ± 11.1 | - | - |
| D_5cc_ | 67.2 ± 9.0 | - | - |
| D_10cc_ | 60.8 ± 6.5 | - | - |
| D_15cc_ | 57.7 ± 5.8 | - | - |
| D_20cc_ | 56.7 ± 5.7 | - | - |

Abbreviations: EQD2 = equivalent dose in the 2-Gy fraction (α/β values of 10 and 3 were applied for the tumor and normal tissues, respectively); D_point A_ = dose to point A; D_100_ and D_90_ = minimal target dose and dose received by at least 90% of the CTV, respectively; D_BP_ = doses to the reference points for the bladder; others are same as in Table 2.

^*^The data for the rectum were calculated from the outer contoured rectal volume.

^†^The data for the rectum were calculated from the rectal wall histogram.

Table s2. Correlation between the rectosigmoid mucosal change (RMC) score and late rectal complication (LRC) grade

|  | ^†^LRC grade | | | |  |
| --- | --- | --- | --- | --- | --- |
| ^*^RMC score | Grade 0 | Grade 1 | Grade 2 | ≥ Grade 3 | ^‡^*p*-value |
| 1 | 24 | 6 | 0 | 0 | < 0.001 |
| 2 | 14 | 6 | 0 | 0 | (*r* = 0.728) |
| 3 | 2 | 2 | 18 | 0 |  |
| 4 | 0 | 0 | 1 | 0 |  |
| 5 | 0 | 1 | 2 | 1 |  |

^*^Endoscopic scoring system .

^†^Radiation Therapy Oncology Group (RTOG) late radiation morbidity scoring criteria .

^‡^Spearman’s rank correlation test.
